# Freshwater microalgae harvested via flocculation induced by pH decrease

**DOI:** 10.1186/1754-6834-6-98

**Published:** 2013-07-09

**Authors:** Jiexia Liu, Yi Zhu, Yujun Tao, Yuanming Zhang, Aifen Li, Tao Li, Ming Sang, Chengwu Zhang

**Affiliations:** 1 Department of Chemistry, Jinan University, Tianhe District, Guangzhou 510632, China; 2 Research Center of Hydrobiology, Jinan University, Tianhe District, Guangzhou 510632, China

**Keywords:** Microalgae, Harvest, Flocculation, pH decrease, Recycle

## Abstract

**Background:**

Recent studies have demonstrated that microalga has been widely regarded as one of the most promising raw materials of biofuels. However, lack of an economical, efficient and convenient method to harvest microalgae is a bottleneck to boost their full-scale application. Many methods of harvesting microalgae, including mechanical, electrical, biological and chemical based, have been studied to overcome this hurdle.

**Results:**

A new flocculation method induced by decreasing pH value of growth medium was developed for harvesting freshwater microalgae. The flocculation efficiencies were as high as 90% for *Chlorococcum nivale*, *Chlorococcum ellipsoideum* and *Scenedesmus sp.* with high biomass concentrations (>1g/L). The optimum flocculation efficiency was achieved at pH 4.0*.* The flocculation mechanism could be that the carboxylate ions of organic matters adhering on microalgal cells accepted protons when pH decreases and the negative charges were neutralized, resulting in disruption of the dispersing stability of cells and subsequent flocculation of cells. A linear correlation between biomass concentration and acid dosage was observed. Furthermore, viability of flocculated cells was determined by Evans Blue assay and few cells were found to be damaged with pH decrease. After neutralizing pH and adding nutrients to the flocculated medium, microalgae were proved to maintain a similar growth yield in the flocculated medium comparing with that in the fresh medium. The recycling of medium could contribute to the economical production from algae to biodiesel.

**Conclusions:**

The study provided an economical, efficient and convenient method to harvest fresh microalgae. Advantages include capability of treating high cell biomass concentrations (>1g/L), excellent flocculation efficiencies (≥ 90%), operational simplicity, low cost and recycling of medium. It has shown the potential to overcome the hurdle of harvesting microalgae to promote full-scale application to biofuels from microalgae.

## Background

Energy is of vital importance to society and human. Biomass energy, as a green and renewable resource, has been considered to be one of the best ways to solve the global energy crisis [1,2]. Microalga is an economical and potential raw material of biomass energy [3], because it does not require a large area of land for cultivation, exhibits short growth period, possesses a high growth rate and contains more high-lipid materials than food crops [4-6]. In general, an algal biomass production system includes growing microalgae in an environment that favors accumulation of target metabolites and recovery of the microalgal biomass for downstream processing [7]. However, due to the small size (5~50 μm), negative surface charge (about -7.5~-40 mV) and low biomass concentration (0.5~5 g/L) of microalgal cells, harvesting microalgal biomass from growth medium is a challenge [8], which accounts for more than 30% of the total production cost from algae to biodiesel [9,10]. Therefore, it is necessary to develop effective and economic technologies for harvesting process.

There are currently several harvesting methods, including mechanical, electrical, biological and chemical based [11]. In mechanical based methods, microalgal cells are harvested by mechanical external forces,such as centrifugation [12], filtration [13], sedimentation [14], dissolved air flotation [15,16] and usage of attached algae biofilms and ultrafiltration membranes [17,18]. Electrical based methods are based on electrophoresis of the algae cells [19,20]. Because of the negative charge of microalgae cells, they can be concentrated by being moved in an electric field [16,21]. Biological based methods are flocculation caused by extracellular polymeric substance such as polysaccharides and proteins, originating from microalgae and microorganism [22]. Chemical based methods mainly refer to chemical flocculation induced by inorganic and organic flocculants. Electrolytes and synthetic polymers are typically utilized [23].

Either physical external forces or chemical flocculantshave been employed in the aforementioned harvesting methods. A few other researchers harvested microalgae by regulating the properties of growth medium. Yahi [24], Vandamme [25] and our previous work [26] have reported that increasing pH value of growth medium induced the hydrolysis of multivalence metallic ions in the growth medium to form metallic hydroxide precipitate, which coagulated microalgal cells by sweeping flocculation and charge neutralization. However, harvesting microalgae *via* regulating the properties of microalgal cells has not been reported. Microalgal cells receive their charge and exhibit dispersing stability from ionization of certain functional groups, such as carboxyl groups on their cell surface [27]. The ionization of functional groups is highly pH-dependent and pH variation has a significant effect on the physicochemical properties of algal cells. To be specific, if acid is added to decrease pH values, carboxylate ions may receive protons and change into neutral carboxyl groups. In this case, the negative charge of cells may be neutralized, their dispersing stability may be destroyed and subsequently they may agglomerate and settle.

In this study, the efficiency of the flocculation method induced by decreasing pH was evaluated. The effects of key factors including pH, biomass concentration, concentration of metal ions and concentration of released polysaccharide (RPS) that might affect the flocculation efficiency were examined. The detailed mechanism was discussed. The relationship between biomass concentration and acid dosage was studied. The recycling of the flocculated growth medium for cultivation was also investigated.

## Results and discussion

### Flocculation of microalgal cells by pH decrease

**Figure 1:**
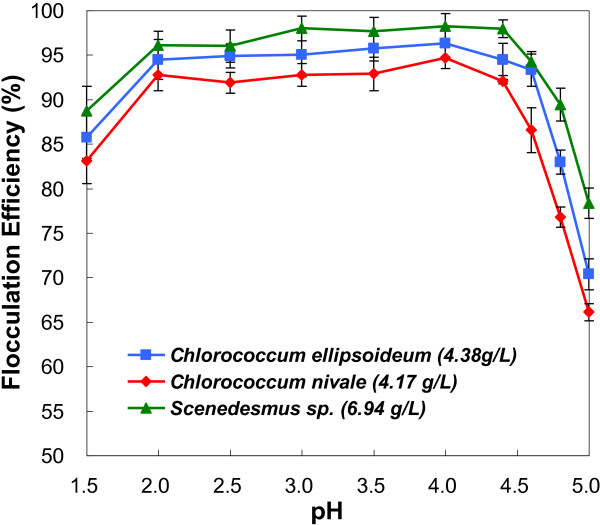
Flocculation efficiencies as a function of pH values for microalgae.

Flocculation efficiencies for the three species of freshwater microalgal cells were studied in terms of pH variations (Figure 1). The microalgal cells began to coagulate when the pH decreased from pH 6.7 to about 5.0 Coagulating of algal cells could still be observed when pH further decreased to 4.5, but the coagulated cells remained suspended in the growth medium. The flocculation efficiencies were relatively low. When the pH value was further decreased to pH 4.0, the cells further coagulated and rapidly subsided within a few minutes, resulting in a higher flocculation efficiencies of 90%. Thus, a flocculation zone was attained when pH was lower than 5.0. The efficiencies reached a maximum at about pH 4.0 and then reached a plateau, with a slight decrease when pH was lower than 2.0. These results showed that flocculation induced by pH decrease is a useful method to harvest the three species of freshwater microalgae. Moreover, the biomass concentrations (>1g/L) in this method were much higher those [23,28] in other harvesting methods (Additional file 1: Table S1). For example, it is 20 times higher than those flocculated using cationic starch [28] and FeCl_3_[29], and about 10 times higher than that flocculated using poly(γ-glutamic acid) [23], indicating that it could be applied to practical uses.

### Mechanisms of flocculation

#### *Role of metal ions in flocculation process*

Earlier studies [25,26,30] reported that multivalent metal ions such as Mg^2+^ and Ca^2+^ played an important role in flocculating microalgae by pH increase. It was found that such metal ions in the growth medium were hydrolyzed to form positive precipitates, which coagulated negative microalgal cells by sweeping flocculation and charge neutralization. To evaluate the role of multivalent metal ions in flocculation induced by pH decrease, their concentrations before and after flocculation for the three microalgae species were measured (Additional file 2: Table S2). Fe^3+^, Mg^2+^ and Ca^2+^ concentrations kept constant before and after flocculation. It suggested that in contrast to the important role of multivalent metal ions in the flocculation by pH increase, they played little part in this flocculation method by pH decrease.

#### *Role of RPS in flocculation process*

**Figure 2:**
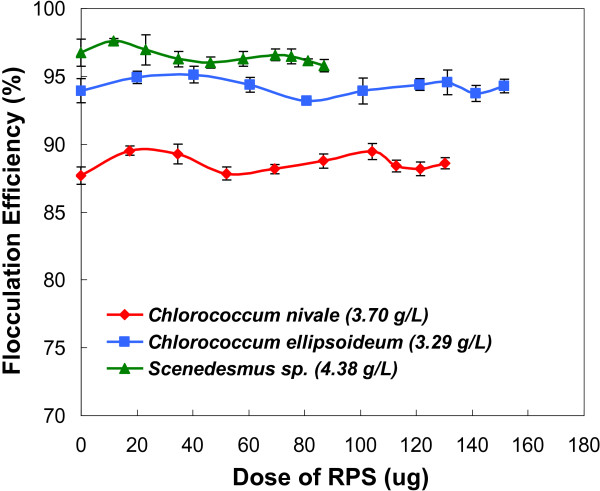
Flocculation tests of microalgae at pH 4.0 in microalgal cultures containing various concentrations of RPS.

It has been reported that many microalgae release large amount of RPS during growth [31], and most RPS can interfere with flocculation due to their complexation with multivalent metal ions [26,32]. However, some RPS are helpful to flocculation owing to the bridging mechanism [33,34]. All of the studied microalgae here released large amount of RPS which was measured and listed in Additional file 3: Table S3. In order to investigate whether RPS was a promoter or hindrance to this flocculation process, the flocculation efficiencies for microalgae were studied in terms of RPS dosages. The presence of RPS caused negligible changes in the flocculation efficiencies for the three microalgae species, suggesting that RPS has little influence (neither a promoter nor a hindrance) on this flocculation process (Figure 2). As the detrimental effect of RPS on microalgal flocculation result from the complexation of RPS with multivalent metal ions, the little influence of RPS on flocculation efficiencies further testified that multivalent metal ions played little part in this flocculation process.

### Mechanism of flocculation

As metal ions and RPS played little part in the flocculation process, mechanism of flocculation might be with the physical-chemical properties of microalgal cells. The most important characteristic of microalgal cells is their surface charge [27]. Thus, zeta potentials of microalgae during flocculation were determined to explore the mechanism.

**Figure 3:**
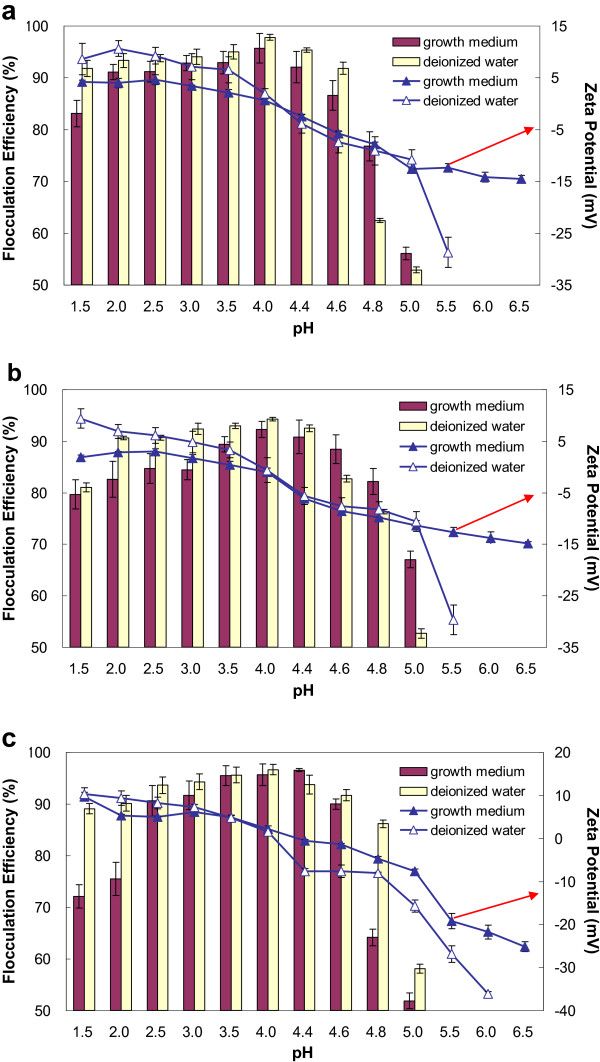
**Zeta potentials and flocculation efficiencies as function of pH values of growth medium and deionized water: a) *Chlorococcum nivale *(3.02 g/L); b) *Chlorococcum ellipsoideum *(3.26 g/L); c) *Scenedesmus *sp. (4.32 g/L).**

Zeta potentials and flocculation efficiencies were both pH dependant (Figure 3). From pH 6.5 to 4.0, zeta potentials showed a sharp increase to approximately 0 mV and the corresponding flocculation efficiencies greatly increased to the maximum with pH decrease.

It has been reported that organic matters adhering on microalgal cell surfaces contain carboxyl groups and amino groups. The amounts of the groups and their pKa values were calculated and listed in Additional file 4: Table S4 The cells usually receive their charge and exhibit dispersing stability from ionization of carboxyl groups into carboxylate ions [27]. The concentrations of the functional groups as a function of pH value were calculated (Additional file 5: Figure S1). For pH>6.0, the microalgae surface charge is dominated by negatively charged carboxylate ions and neutral amine groups. As pH decreased, carboxylate ions would accept protons [Eq. (1)] [29]. Then, the surface charge of the cells reduced and the cells became instable in growth medium and coagulated to form big flocs. When the surface charge was totally neutralized at pH 4.0, flocculation efficiencies reached the maximum.

However, from pH 4.0 to 1.5, zeta potentials continuously increased while the corresponding flocculation efficiencies slightly decreased. It might be because that the concentrations of the neutral carboxyl groups increased sharply while the concentrations of the positively charged amine groups (-NH_3_^+^) remained constant [Eq. (2)]. This caused the zeta potential increasing, and the positive surface charge made the microalgal cells resuspend so flocculation efficiencies slightly decreased.

The above proposed mechanism involved only protons and functional groups. But, growth medium also contains large amounts of metal salts and extracellular organic matter (EOM). The contributions of metal salts and EOM to the mechanism were evaluated. The average zeta potentials and flocculation efficiencies in deionized water as a function of pH value were also shown in Figure 3. Compared with those in growth medium, the variation trends of zeta potentials and flocculation efficiencies were extremely similar. From pH 6.5 to 1.5, zeta potentials continuously increased but flocculation efficiencies firstly increased then decreased. pH 4.0 was the transiting point, at which flocculation efficiencies reached peak and the cells were electrically neutral. The results further confirmed that flocculation was induced only by neutralizing cells surface charges with protons while metal salts and EOM had no contribution to the mechanism.

(1) $${}^{-}\mathrm{OOC}‒\mathrm{cell}‒NH_{2}+H^{+}\to \mathrm{HOOC}‒\mathrm{cell}‒NH_{2}$$

(2) $$\mathrm{HOOC}-\mathrm{cell}-{\mathrm{NH}}_{2}+H^{+}\to \mathrm{HOOC}-\mathrm{cell}-{\mathrm{NH}}_{3}{}^{+}$$

### Flocculation dependence on microalgae biomass concentration

**Figure 4:**
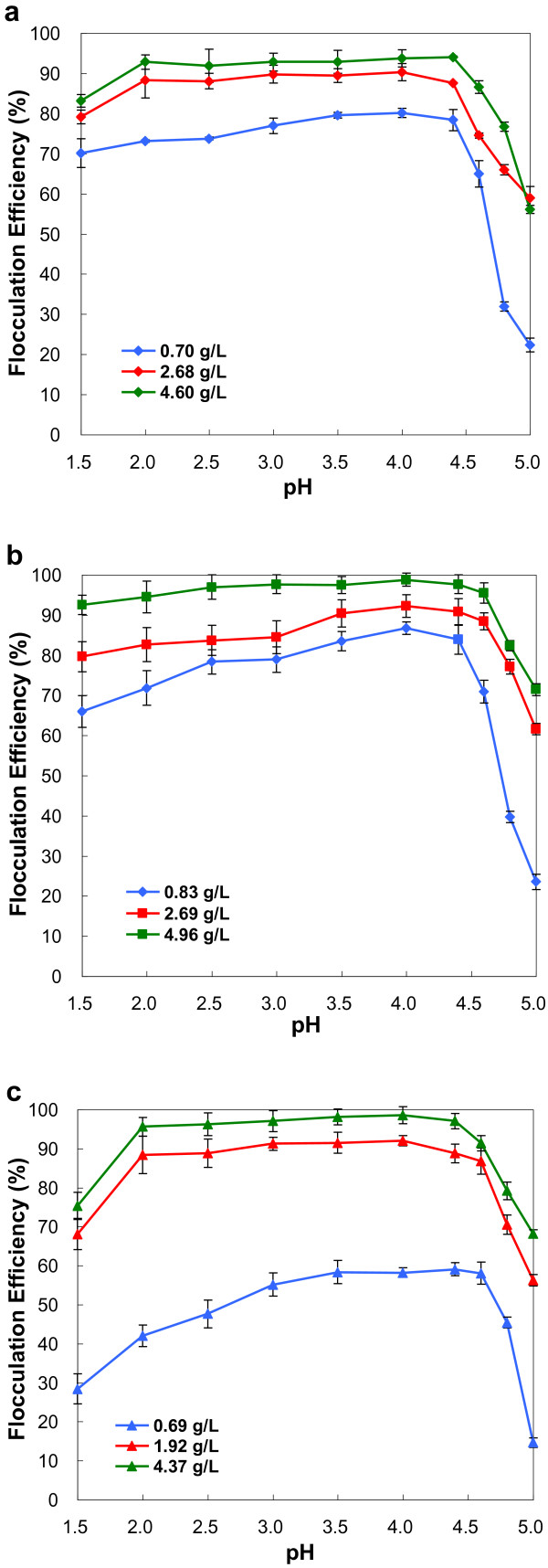
**Flocculation efficiencies of microalgae with different biomass concentrations as a function of pH values. a)***Chlorococcum nivale*; **b)***Chlorococcum ellipsoideum*; **c)***Scenedesmus* sp.

**Figure 5:**
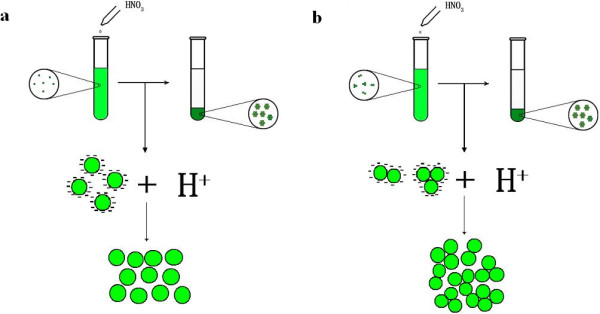
**Schematic diagram of the possible mechanisms during flocculation process in different growth stages of microalgae. a)** early growth stage; **b)** exponential growth phase and stationary phase.

In our previous report [26], microalgae biomass concentrations have effects on the flocculation efficiencies induced by pH increase and the efficiencies decreased considerably with the increase of biomass concentrations. However, in this study, the flocculation efficiencies at the same pH value for the microalgae were all increased with the increase of biomass concentrations (Figure 4). Low biomass concentrations correspond to lag phase, while high biomass concentrations correspond to exponential growth phase and stationary phase. During lag phase, microalgae are unicells. But during exponential growth phase and stationary phase, microalgal cells usually coagulate by threes and fours to form cell clusters. As the surface charge of cells are generally neutralized, it is easier for the heavier cell clusters than the unicells to settle (Figure 5). So, the flocculation efficiencies increased with the increase of biomass concentrations.

### Relationship between biomass concentration and amount of HNO_3_

**Figure 6:**
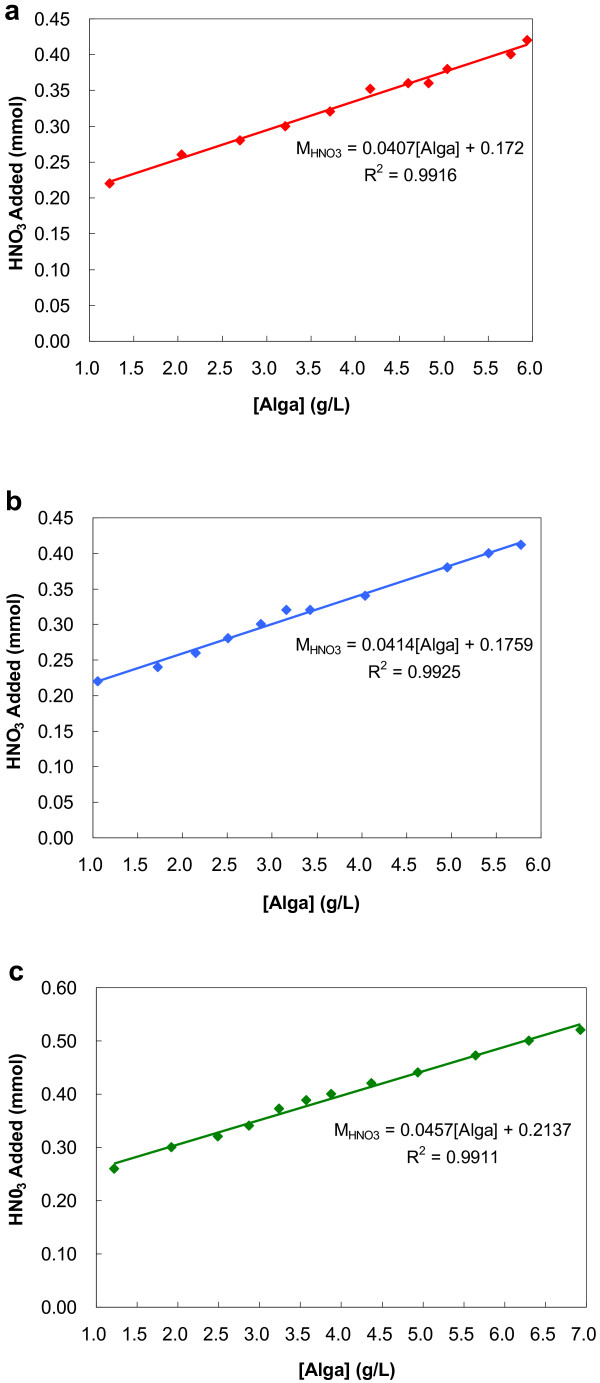
**Minimum amounts of HNO**_**3 **_**needed for 90% flocculation efficiencies as function of biomass concentrations. a)***Chlorococcum nivale*; **b)***Chlorococcum ellipsoideum*; **c)***Scenedesmus* sp*.*

As mentioned above, flocculation efficiencies for the three microalgae species were increased with the increase of biomass concentrations. However, the minimum dosages of HNO_3_ resulting in the same flocculation efficiencies also rose accordingly. Hence, the biomass concentration is a very important parameter affecting the optimal dosage of HNO_3_. Experiments were run at different biomass concentrations to determine the correlation between biomass concentration and HNO_3_ dosage (Figure 6). A linear correlation was thus observed, which was expressed by the following equations [Eqs. (3), (4) and (5) for *Chlorococcum nivale* and *Chlorococcum ellipsoideum* and *Scenedesmus sp*.] Hence, in practical uses, acid dosage needed for flocculating microalgae can be calculated according to biomass concentration and their relationship.

(3) $$M_{\mathrm{HNO}3}=0.0407\left[\mathrm{Alga}\right]+0.172$$

(4) $$M_{\mathrm{HNO}3}=0.0414\left[\mathrm{Alga}\right]+0.1759$$

(5) $$M_{\mathrm{HNO}3}=0.0457\left[\mathrm{Alga}\right]+0.2137$$

### Cells viability during pH decrease process

**Figure 7:**
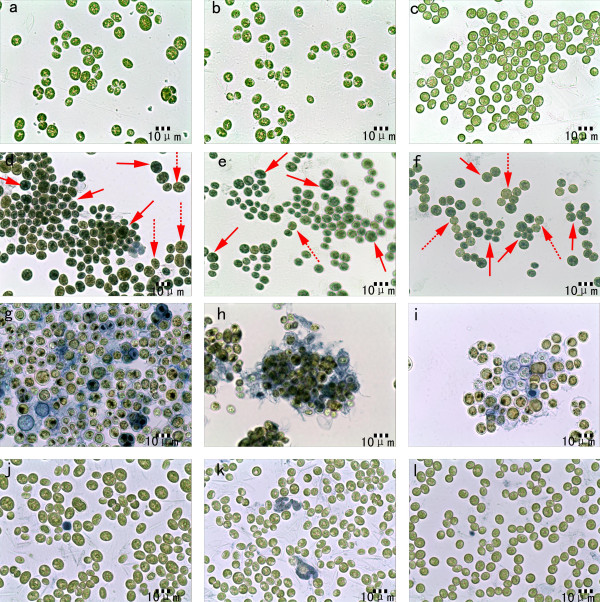
**Microscopic pictures of microalgal cells: controlled cells: a) *Chlorococcum nivale*; b) *Chlorococcum ellipsoideum*; c) *Scenedesmus *sp*.*; cells heated at 121 and incubated in 1% Evans’ blue solution for 3 h: d) *Chlorococcum nivale*; e) *Chlorococcum ellipsoideum*; f) *Scenedesmus *sp*.*; cells flocculated by adjusting pH value of growth medium to 0.5 with nitric acid and incubated in 1% Evans’ blue solution for 3 h: g) *Chlorococcum nivale*; h) *Chlorococcum ellipsoideum*; i) *Scenedesmus *sp*.*; cells flocculated by adjusting pH value of growth medium to 3.5 with nitric acid and incubated in 1% Evans’ blue solution for 3 h: j) *Chlorococcum nivale*; k) *Chlorococcum ellipsoideum*; l) *Scenedesmus *sp*.***

Viability of microalgal cells was determined by the Evans blue assay and the cells seemed to be very resistant to relatively low pH values (6.0-3.0). A positive control is also provided. As shown in Figures 7a, 7b and 7c, the controlled cells are light green and the yellow liquid in the cells can be seen clearly. However, for the cells heated at 121 in Figures 7d, 7e and 7f, the dead cells (solid arrows) are dark green which were dyed by Evans blue and the yellow liquid in the cells are not visible. While the alive cells (dash arrows) are light green and similar to the cells shown in Figures 7a, 7b and 7c. As for the cells flocculated by adjusting pH value of growth medium to 0.5 with nitric acid (Figure 7g, 7h and 7i), the dead cell nuclei turned black, the cytoplasm turned green-yellow and the materials surrounding the cells were dyed blue . The cells flocculated by adjusting pH value of growth medium to 3.5 in Figures 7j, 7k and 7l, are similar to the controlled cells in Figure 7a, 7b and 7c, except that few cells were dyed blue. The above results indicated there were no cell lysis and the cell walls were intact. Thus, the cells were not damaged during the process of pH decreasing to 3.5.

### Recycling of flocculated culture medium for cultivation

**Figure 8:**
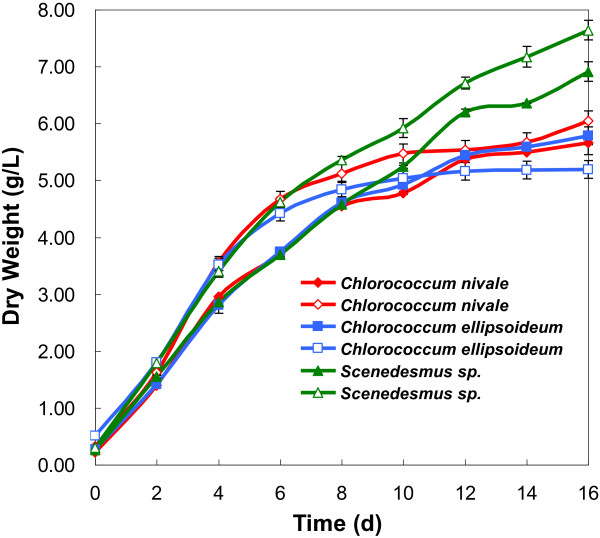
Growth curves of microalgae: fresh medium (filled points); flocculated medium (empty points).

Ideally, medium recovered from flocculation could be recycled for next cultivation. The problem with medium recycling is that residual flocculant such as ferric salts and aluminum salts can cause contamination, which eventually cause environmental problems and a great loss of water [35]. However, in this flocculation method induced by pH decrease, since no flocculants were used and the medium was not contaminated, the growth medium after flocculation might be recycled by neutralizing pH and then adding nutrients. The product of neutralizing pH of flocculated BG-11 medium with NaOH was NaNO_3_, which was a necessary nutrient. So, the recycling of flocculated medium could minimize the cost of nutrients and demand for water. In this respect, the possibility of recycling the flocculated medium was examined. Some microalgal cells flocculated were cultivated in the recycled culture solution and the biomass as a function of growth phase was shown in Figure 8. It was observed that the biomass of each microalgal species cultivated in the recycled growth medium was close to that cultivated in the fresh medium, indicating the flocculated medium could be successfully recycled for cultivation. The fact that the flocculated microalgal cells could be recultivated further suggested that there was no cell lysis during the flocculation process and the molecular function and structure of the photosynthetic apparatus were not affected.

### Comparison with other harvesting methods

The flocculation method presented here is simple and effective. The traditional harvesting methods, such as ultrasound, flotation, centrifugation and filtration, were also successfully applied to a range of microalgal species. But they are energy-intensive and cost-intensive [36]. Moreover, microalgae can also be harvested by using flocculants, including inorganic and organic flocculants. Inorganic flocculants such as aluminum salts, ferric salts and zinc salts were often used, but caused an environmental problem and a great loss of water due to the contamination of residual inorganic flocculants. Organic flocculants, such as cationic polyacrylamide, cationic starch and chitosan, are biodegradable and low toxic [37], but are of high cost owing to their high prices ($US10 per kilogram for chitosan and $US 1~3 per kilogram for cationic starch [28]). In this study, microalgae were flocculated induced by HNO_3_. In contrast, HNO_3_, is inexpensive and about $US 0.28 per kilogram. And what’s more, HNO_3_ does not contaminate growth medium, which can be recycled to reduce not only the cost and the demand for water, but also the extra operational costs for reusing growth medium. Additionally, comparison of the cost of cultivation and flocculation for per kilogram microalgae has been listed in Additional file 6: Table S5. Thereby this method is helpful to lower the production cost from algae to biodiesel.

## Conclusions

A flocculation method induced by pH decrease was developed for harvesting freshwater microalgae. Advantages include capability of treating high cell biomass concentrations (> 1g/L), excellent flocculation efficiencies ( ≥ 90%), operational simplicity, low cost and recycling of medium. So the method could contribute to the economical production from algae to biodiesel. The mechanism could be that carboxylate ions of organic matters adhering on cells accepted protons with pH decrease, the negative charges were neutralized and then the dispersing stabilities of cells were destroyed. A linear correlation between biomass concentration and acid dosage was also observed. Further work is required to evaluate the application of such a method to the marine microalgae.

## Methods

### Microalgal strains and culture condition

*Chlorococcum nivale* UTEX2765, *Chlorococcum ellipsoideum* UTEX 972 and *Scenedesmus* sp. JNU-49 were used in this study. All strains were kindly supplied by Laboratory of Microalgal Bioenergy & Biotechnology, Research Center of Hydrobiology at Jinan University. The microalgal cells are all spherical and the average diameters are 6.2±1.17 μm (*Chlorococcum nivale*), 8.5±1.43 μm (*Chlorococcum ellipsoideum*) and 7.7±0.80 μm (*Scenedesmus* sp.), respectively. All algae were grown in a BG-11 medium containing the following components: NaNO_3_ (1.5g L^-1^) ; K_2_HPO_4_·3H_2_O (40mg L^-1^) ; MgSO_4_·7H_2_O (75 mg L^-1^) ; CaCl_2_·2H_2_O (36 mg L^-1^) ; Na_2_CO_3_ (20 mg L^-1^); FeCl_3_·6H_2_O (3.15 mg L^-1^) ; citric acid (6 mg L^-1^) and 1 ml of microelements composed of H_3_BO_3_ (2.86 mg L^-1^), MnCl_2_·4H_2_O (1.81 mg L^-1^), ZnSO_4_·7H_2_O (0.22 mg L^-1^), Na_2_MoO_4_·2H_2_O (0.39 mg L^-1^),CuSO_4_·5H_2_O (0.08 mg L^-1^), Co(NO_3_)_2_·6H_2_O (0.05 mg L^-1^) in 1000 ml acidified water which included 1 ml concentrated H_2_SO_4_ in 1 L distilled water.

All the microalgae were grown in a 210 mL glass column photobioreactor (Φ3.5×60 cm). Both the BG-11 medium and glass column photobioreactors were sterilized at 121°C for 20 min. The cultures were incubated at 25°C and illuminated using cool-white fluorescent lamps for 24h. The average light intensity at the surface of the glass column photobioreactors was kept at 200 μmol·m^-2^·s^-1^. The cultures were continuously aerated by bubbling air containing 1% CO_2_ (v/v).

### Determination of dry weight

Algae biomass concentrations reported in this study are expressed by algal dry weight. Dry weight was determined by filtering a fixed volume of the algae suspension through a pre-weighed filter with a 0.45 μm porous membrane. Then the filter and algal cells were dried at 105°C for 48 h. Finally, the algal dry weight was calculated by subtracting the mass of the filter from the total mass.

### Flocculation by pH decrease

Flocculation experiments were all run with small volumes of medium (20 mL) distributed in cylindrical glass tubes (40 mL). The pH value of each sample was gradually adjusted by addition of 1M HNO_3_. The glass tube was vortexed thoroughly for 30 s as soon as the pH had been adjusted and allowed to stand at room temperature for 15 min. Then an aliquot of medium was pipetted and used to measure OD_750_ (optical density at the wavelength of 750 nm).

### Determination of flocculation efficiency

The flocculation efficiency of each sample was calculated according to following equation:

$$\mathrm{flocculation}\;\mathrm{efficiency}\;\left(\%\right)=\left(1-A/B\right)\times 100$$

Where A is the OD_750_ of sample and B is the OD_750_ of the reference.

### Zeta potential

A Malvern Zetasizer 2000HSA (Malvern, UK) was utilized to determine the zeta potentials of the system. The zeta potentials of microalgal cells in original growth medium and in deionized water were both measured within a pH range of 1.5~6.0 by adding 1M HNO_3_. Microalgal cells in deionized water were prepared by isolating cells from the growth medium via centrifugation, washing and resuspending cells in deionized water. 1.0mL suspension was pipetted into a cuvette and inserted into the units for zeta potential. Zeta potential was performed in triplicate at room temperature and the average values were then taken into account.

### Role of RPS in flocculation process

The microalgal cells were removed by centrifugation for the purpose of preparing corresponding media containing various concentrations of RPS. The supernatant was filtered through a 0.45 μm porous membrane, followed by a 0.22 μm porous membrane. The filtered supernatant was dialysed against distilled water for 72 h and the dialysed RPS solution was used as stock solution. RPS solution was measured by the phenol-sulfuric acid method [38], using glucose as a standard. To prepare microalgal culture in corresponding media containing various concentrations of RPS, microalgal cells were collected via centrifugation and washed twice with deionized water, and then the cells were resuspended in the growth medium containing various concentrations of RPS which was prepared by diluting the above RPS stock solution with growth medium.

The comparative coagulation tests were performed using microalgal cultures in corresponding media containing various concentrations of RPS so as to investigate the effect of RPS on the flocculation process. The role of RPS was assessed by difference of flocculation efficiency in the presence of various concentrations of RPS compared to that in their absence.

### Viability assay of cells

The viability of cells was tested using 1% Evans Blue dye, which is excluded by viable cells [39]. 1 mL samples of each flocculated medium (after being flocculated via reducing pH of growth medium to desired value at for 2 h) were centrifuged and the supernatant was discarded. Then 100 μL of 1% Evans blue solution was added, and the cells were incubated for 3 h at room temperature. Next, the cells were washed twice using deionized water to remove excessive and unbound dye. Finally, fresh preparations of the centrifuged samples were examined for the viability by an optical microscope. As Evans blue solution diffused in the protoplasm region and stained the cells blue, and cells with broken cell walls appeared blue.

### Recycling of flocculated medium

The flocs and the growth medium were separated after flocculation. The pH of remaining culture medium, named the flocculated medium, was adjusted back to the pH before flocculation by adding the necessary amount of 1M NaOH. After that, nutrients such as K_2_HPO_4_·3H_2_O (40mg L^-1^), MgSO_4_·7H_2_O (75 mg L^-1^), CaCl_2_·2H_2_O (36 mg L^-1^), Na_2_CO_3_ (20 mg L^-1^), FeCl_3_·6H_2_O (3.15 mg L^-1^), citric acid (6 mg L^-1^) and 1 ml of microelements in 1L treated medium, were also added to the flocculated medium. Microalage were cultivated in the fresh medium and the flocculated medium. Comparation of the biomass concentration as a function of growth phase was investigated.

### Other equipments

OD_750_ was measured using a Lambda 45 UV–vis spectrometer (Perkin-Elmer Intruments). Microalgal cells were obtained and sized on an optical microscope (OLYMPUS CX41RF) using a scale graticule. The concentrations of the metal ions in the growth media were determined using inductively coupled-atomic emission spectrometry (ICP-AES).

## Abbreviations

RPS: Released polysaccharide.

## Competing interests

The authors declare that they have no competing interests.

## Authors’ contributions

YZ, YZ, AL, MS and CZ conceived of the study. JL, YZ, YZ, AL and CZ drafted the manuscript. JL and TL carried out microalgal cultivation and evaluated the recycling of flocculated medium. JL and YT carried out all the flocculation experiments and mechanism study. “All authors read and approved the final manuscript”.

## Supplementary Material

Additional file 1: Table S1 Comparison of the biomass concentrations for different methods. Click here for file

Additional file 2: Table S2 Concentrations of metal ions: Fe^3+^, Mg^2+^ and Ca^2+^ (mg/L) in the growth medium before and after flocculation (pH 4.0) for the three microalgae species. Click here for file

Additional file 3: Table S3 The released amounts of RPS for all the studied microalgae in different growth stage. Click here for file

Additional file 4: Table S4 pKa values and associated functional groups for the studied microalgae. Click here for file

Additional file 5: Figure S1 Concentrations of the functional groups on microalgae surface as a function of pH values: **a)***Chlorococcum nivale* (2.07 g/L); **b)***Chlorococcum ellipsoideum* (1.97 g/L); **c)***Scenedesmus sp.* (2.40 g/L). Click here for file

Additional file 6: Table S5 Comparison of the cost of cultivation and flocculation for per kilogram microalgae. Click here for file
