# Supplementary material for: Freshwater microalgae harvested via flocculation induced by pH decrease
Source: Biotechnol Biofuels. 2013 Jul 9;6:98. doi: 10.1186/1754-6834-6-98 (PMC3716916; doi:10.1186/1754-6834-6-98)
Supplement: Additional file 1: Table S1 — Comparison of the biomass concentrations for different methods. [file 1754-6834-6-98-S1.doc]

| **Harvesting method** | **Species** | **Biomass** | **Flocculation efficiency** | **Reference** | **Number in the reference list of the revised manuscript** |
| --- | --- | --- | --- | --- | --- |
| Cationic starch | *Parachlorella, Scenedesmus* | 0.075~0.35 g L^-1^ | 90%(at the optimal dose) | *J Appl Phycol* , 2010 **22**: 525–530 | 28 |
| Poly(γ-glutamic acid) | *Chlorella vulgaris,* | 0.57g L^-1^ | >90% | *Bioresource Technol*, 2012 **112**: 212–220 | 23 |
|  | *Chlorella protothecoides* | 0.60g L^-1^ |  |  |  |
| FeCl_3_ | *Chlorella zofingiensis* | 0.05~0.12g L^-1^ | >90% | *Biotechnol Bioeng*, 2012, **109:** 493-501 | 29 |
| This study | *Chlorococcum nivale* | 1.0~6.0 g L^-1^ | >90% |  |  |
|  | *Chlorococcu ellipsoideum* | 1.0~6.0 g L^-1^ |  |  |  |
|  | *Scenedesmus* sp. | 1.0~7.0 g L^-1^ |  |  |  |
