# Supplementary material for: Freshwater microalgae harvested via flocculation induced by pH decrease
Source: Biotechnol Biofuels. 2013 Jul 9;6:98. doi: 10.1186/1754-6834-6-98 (PMC3716916; doi:10.1186/1754-6834-6-98)
Supplement: Additional file 2: Table S2 — Concentrations of metal ions: Fe3+, Mg2+ and Ca2+ (mg/L) in the growth medium before and after flocculation (pH 4.0) for the three microalgae species. [file 1754-6834-6-98-S2.doc]

| **Algae** | **Before Flocculation**  **(mg/L)** | | |  | | | **After Flocculation**  **(mg/L)** | | |
| --- | --- | --- | --- | --- | --- | --- | --- | --- | --- |
|  | **Fe^3+^** | **Mg^2+^** | **Ca^2+^** | |  | **Fe^3+^** | | **Mg^2+^** | **Ca^2+^** |
| ***Chlorococcum nivale***  (2.69 g/L) | 0.005 | 5.29 | 10.45 | |  | 0.055 | | 5.40 | 10.09 |
| ***Chlorococcum ellipsoideum***  (4.10 g/L) | 0.015 | 5.27 | 10.64 | |  | 0.075 | | 5.41 | 10.17 |
| ***Scenedesmus* sp.**  (4.33 g/L) | 0.025 | 6.42 | 10.39 | |  | 0.021 | | 6.40 | 10.22 |
