# Supplementary material for: Freshwater microalgae harvested via flocculation induced by pH decrease
Source: Biotechnol Biofuels. 2013 Jul 9;6:98. doi: 10.1186/1754-6834-6-98 (PMC3716916; doi:10.1186/1754-6834-6-98)
Supplement: Additional file 3: Table S3 — The released amounts of RPS for all the studied microalgae in different growth stage. [file 1754-6834-6-98-S3.doc]

| **Stage**  **Algae** | Early growth  stage | Exponential growth phase | Stationary  phase |
| --- | --- | --- | --- |
| ***Chlorococcum nivale*** | 34.98 mg/L | 41.06 mg/L | 35.42 mg/L |
| ***Chlorococcum ellipsoideum*** | 51.91 mg/L | 59.29 mg/L | 40.63 mg/L |
| ***Scenedesmus* sp.** | 30.21mg/L | 35.42 mg/L | 34.11 mg/L |
