# Supplementary material for: Freshwater microalgae harvested via flocculation induced by pH decrease
Source: Biotechnol Biofuels. 2013 Jul 9;6:98. doi: 10.1186/1754-6834-6-98 (PMC3716916; doi:10.1186/1754-6834-6-98)
Supplement: Additional file 4: Table S4 — pKa values and associated functional groups for the studied microalgae. [file 1754-6834-6-98-S4.doc]

| **Species** | **-COOH** | |  | **-NH_3_^+^** | |
| --- | --- | --- | --- | --- | --- |
|  | **pKa** | **Density**  (10^-3^ mmol/g  dry weight) |  | **pKa** | **Density**  (10^-3^ mmol/g  dry weight) |
| *Chlorococcum nivale* | 3.91 | 4.365 |  | 9.88 | 2.042 |
| *Chlorococcum ellipsoideum* | 3.98 | 1.277 |  | 9.12 | 0.966 |
| *Scenedesmus* sp. | 4.24 | 1.915 |  | 8.58 | 1.216 |
