# Supplementary figures and images for: Freshwater microalgae harvested via flocculation induced by pH decrease
Source: Biotechnol Biofuels. 2013 Jul 9;6:98. doi: 10.1186/1754-6834-6-98 (PMC3716916; doi:10.1186/1754-6834-6-98)

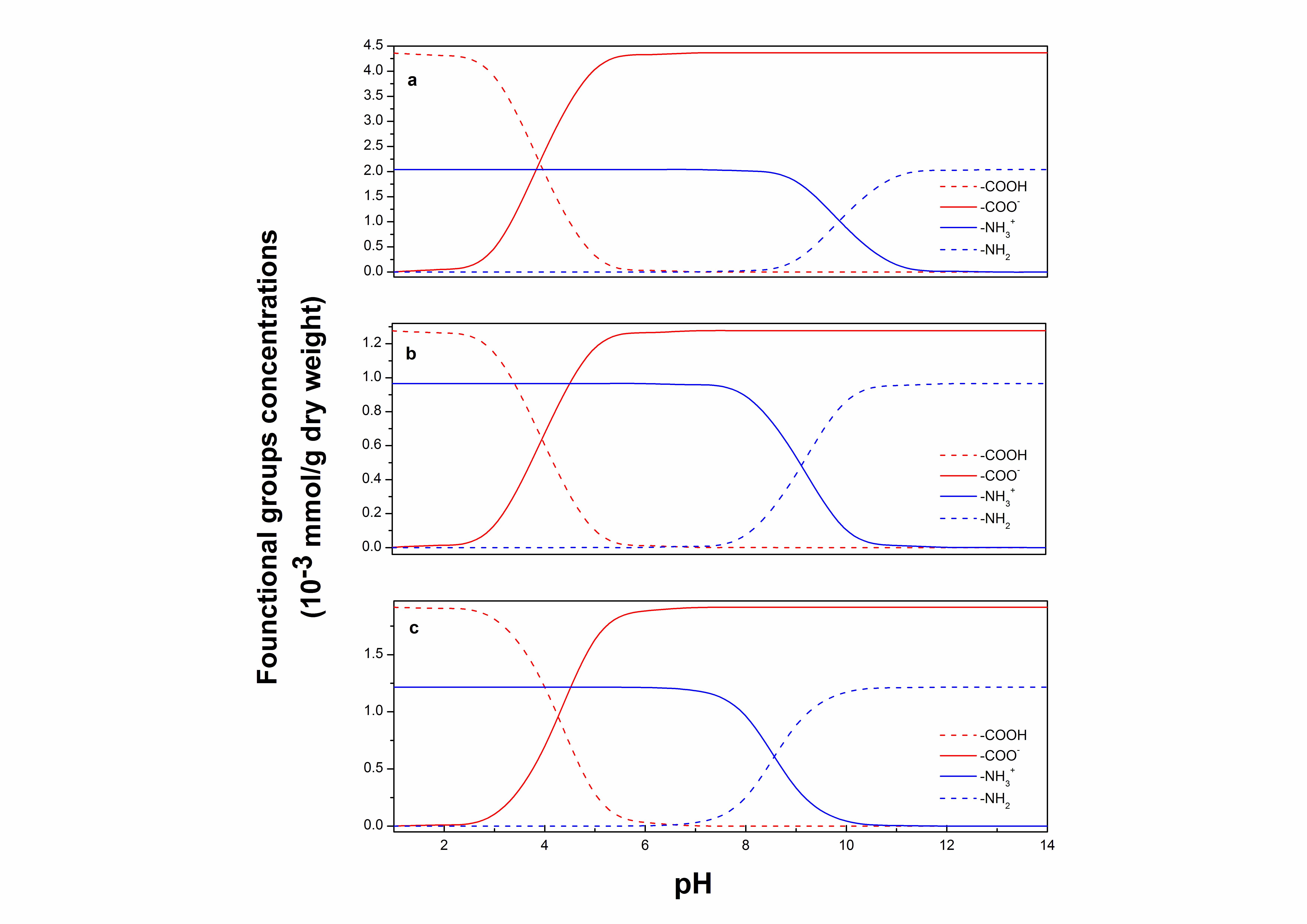

Supplement: Additional file 5: Figure S1 — Concentrations of the functional groups on microalgae surface as a function of pH values: a)Chlorococcum nivale (2.07 g/L); b)Chlorococcum ellipsoideum (1.97 g/L); c)Scenedesmus sp. (2.40 g/L). [file 1754-6834-6-98-S5.jpeg]
