# Supplementary material for: Freshwater microalgae harvested via flocculation induced by pH decrease
Source: Biotechnol Biofuels. 2013 Jul 9;6:98. doi: 10.1186/1754-6834-6-98 (PMC3716916; doi:10.1186/1754-6834-6-98)
Supplement: Additional file 6: Table S5 — Comparison of the cost of cultivation and flocculation for per kilogram microalgae. [file 1754-6834-6-98-S6.doc]

| **Cost**  **Algae** | **Cultivation cost**  **NaNO_3_（$US）** | **Flocculation cost**  **HNO_3_（$US） NaOH（$US）** | |
| --- | --- | --- | --- |
| ***Chlorococcum nivale*** | 0.118 | 0.0612 | 0.0180 |
| ***Chlorococcum ellipsoideum*** | 0.118 | 0.0624 | 0.0184 |
| ***Scenedesmus* sp.** | 0.102 | 0.0717 | 0.0211 |
